# Effects of Vitamin D on Airway Epithelial Cell Morphology and Rhinovirus Replication

**DOI:** 10.1371/journal.pone.0086755

**Published:** 2014-01-24

**Authors:** Rebecca A. Brockman-Schneider, Raymond J. Pickles, James E. Gern

**Affiliations:** 1 Department of Pediatrics and Medicine, University of Wisconsin, Madison, Madison, Wisconsin, United States of America; 2 Department of Microbiology and Immunology, University of North Carolina, Chapel Hill, Chapel Hill, North Carolina, United States of America; Maastricht University Medical Center (MUMC), The Netherlands

## Abstract

Vitamin D has been linked to reduced risk of viral respiratory illness. We hypothesized that vitamin D could directly reduce rhinovirus (RV) replication in airway epithelium. Primary human bronchial epithelial cells (hBEC) were treated with vitamin D, and RV replication and gene expression were evaluated by quantitative PCR. Cytokine/chemokine secretion was measured by ELISA, and transepithelial resistance (TER) was determined using a voltohmmeter. Morphology was examined using immunohistochemistry. Vitamin D supplementation had no significant effects on RV replication, but potentiated secretion of CXCL8 and CXCL10 from infected or uninfected cells. Treatment with vitamin D in the form of 1,25(OH)_2_D caused significant changes in cell morphology, including thickening of the cell layers (median of 46.5 µm [35.0–69.0] vs. 30 µm [24.5–34.2], p<0.01) and proliferation of cytokeratin-5-expressing cells, as demonstrated by immunohistochemical analysis. Similar effects were seen for 25(OH)D. In addition to altering morphology, higher concentrations of vitamin D significantly upregulated small proline-rich protein (SPRR1β) expression (6.3 fold-induction, p<0.01), suggestive of squamous metaplasia. Vitamin D treatment of hBECs did not alter repair of mechanically induced wounds. Collectively, these findings indicate that vitamin D does not directly affect RV replication in airway epithelial cells, but can influence chemokine synthesis and alters the growth and differentiation of airway epithelial cells.

## Introduction

Vitamin D_3_ is a fat soluble hormone obtained primarily through sun exposure, and to a lesser extent from the diet. Activation of vitamin D_3_ requires two sequential hydroxylation steps. The first takes place in the liver, where the enzyme 25-hydroxylase converts vitamin D_3_ to 25-hydroxyvitamin D_3_ (25(OH)D): the circulating, pre-hormone form of the vitamin. Subsequently, 25-hydroxyvitamin D_3_ is converted to 1,25-dihydroxyvitamin D_3_ (1,25(OH)_2_D) through the action of the enzyme 1α-hydroxylase. This enzyme has traditionally been associated with the kidney, but has since been found elsewhere in the body, including in bronchial epithelial cells [1]. This latter compound, 1,25(OH)_2_D, represents the active, hormonal form of vitamin D_3_. Recent evidence indicates that vitamin D_3_ is involved in regulating genes that play a role in immunity [2], [3]. In addition, vitamin D acts on epithelial cells to stimulate the secretion of cathelicidin and other peptides that protect against infections with bacteria and enveloped viruses [4], [5].

There is clinical evidence that vitamin D levels are inversely related to respiratory illnesses, as well as exacerbations of asthma, which are often provoked by viruses such as rhinoviruses (RV) [6], [7]. The respiratory epithelium plays a critical role in defending against RVs through the activation of antiviral pathways, and the secretion of chemokines that recruit effector cells to the site of infection. In addition, the barrier function of airway epithelium also protects against RV infection; disruption of an intact epithelial layer in vitro significantly enhances RV replication [8]. Collectively, these findings suggest that vitamin D could inhibit the growth of RVs, either directly or indirectly by influencing the growth and/or differentiation of the airway epithelium.

To test this hypothesis, we added vitamin D to primary cultures of human bronchial epithelial cells (hBEC), and measured effects of vitamin D on RV replication, hBEC morphology and growth, epithelium integrity by monitoring transepithelial resistance (TER), and alterations in select gene expression levels. Two different models, involving addition of vitamin D to cells either during or following differentiation, were enlisted to investigate effects of vitamin D on airway epithelial cells.

Here we report that vitamin D does not directly affect RV replication in airway epithelial cells. Vitamin D does induce the synthesis of two chemokines, CXCL8 and CXCL10, showing an additive effect in conjunction with viral infection. In the course of conducting these experiments, it was incidentally noted that vitamin D has significant effects on the morphology of cultured cell layers, and higher concentrations of vitamin D produce changes similar to those of vitamin A deficiency.

## Materials and Methods

### Ethics Statement

Primary hBECs were derived from residual human surgical specimens from healthy lung donors. The protocol was approved by the University of Wisconsin’s Institutional Review Board, which waived the need for consent.

### Differentiation of hBECs with Vitamin D Metabolites

Passage 1 primary hBECs were prepared in permeable membrane supports (12-well; 0.4 µm pore size, Corning Incorporated, Corning, NY) as previously described [8], [9]. After 24 hrs, BEGM medium was removed from both the upper and lower chamber, and medium in the lower chamber only was replaced with 1 mL of air-liquid interface (ALI) medium, consisting of a 50∶50 mixture of BEGM and DMEM (Mediatech, Manassas, VA) that had been supplemented with all the BEGM additives (Lonza) except retinoic acid. To this mixture, fresh all-*trans* retinoic acid (50 nM, Sigma-Aldrich) was added, along with either 1,25(OH)_2_D or 25(OH)D (0.1, 1, 10, and 100 nM, Sigma-Aldrich). Since serum levels of 30–80 ng/mL (75–200 nM) 25(OH)D are considered optimal for health, with levels of 1,25(OH)_2_D being dependent upon the degree of localized synthesis, the range of concentrations used in our experiments did not exceed the physiologic range [1]. Medium with retinoic acid was replaced daily during the first week, and then every 2–3 days. Transepithelial resistance measurements were made using a voltohmmeter.

### Treatment of Fully Differentiated hBECs with 1,25(OH)_2_D

Cultures were prepared as above, with 1,25(OH)_2_D withheld until differentiated cultures were established (4 weeks), after which 10 nM 1,25(OH)_2_D was added to medium in lower compartment.

### Immunohistochemistry

Differentiated cell layers were fixed with 10% phosphate-buffered formalin, and were stained with hemotoxylin and eosin, and alcian blue (VA Hospital, Madison, WI), as well as acetylated alpha tubulin (mouse mAb, Abcam, Cambridge, MA) and fibroblast specific protein (polyclonal rabbit anti-FSP1/S100A4, Millipore, Temecula, CA; UW-Madison’s Carbone Cancer Center Experimental Pathology Laboratory). Staining for MUC5AC, smooth muscle actin (SMA), and cytokeratin (CK 5/6) was performed by US Labs (Brentwood, TN). Average thicknesses of cell layers were determined as follows: sequential photos were taken along the length of each stained section, the area (µm^2^) and length (µm) of the section in each photo was measured using ImageJ software, and area/length (µm) calculations represented the average thickness of the layers.

### Wounding of Cell Layers

Differentiated cell layers were wounded with a sterile P200 pipet tip [8]. Cell layers were washed to remove loose cells, and wound areas in photos were measured using SigmaScan Pro 5 image measurement software.

### Infection of Cells

Differentiated cell layers were incubated with purified RV-A16 (5×10^6^ pfu/chamber, MOI = 10). Experiments that examined lower inoculating doses included 10-fold dilutions of the virus (5×10^5^ pfu/chamber, MOI = 1; 5×10^4^ pfu/chamber, MOI = 0.1). Cultures were incubated for 4 hrs at 34°C and then cell layers were washed 3 times. Cultures were then incubated for an additional 24 hrs (34°C, 5% CO_2_), after which medium in the lower chamber was cryopreserved for future analysis, and cell lysates were prepared.

### Quantitative PCR

RNA was extracted from cell lysates with Trizol (Invitrogen, Carlsbad, CA), treated with DNase (Promega, Madison, WI) for 15 minutes at 37°C, and cleaned up with a MinElute kit (Qiagen, Valencia, CA). cDNA was synthesized using a TaqMan RT kit (Applied Biosystems, Foster City, CA). RV RNA was measured using real-time PCR (Prism 7000, Applied Biosystems, Foster City, Calif) with primers and probes described previously [10]. The standard curve was developed by extracting a known concentration of RV16 (1×10^3^–1×10^8^ PFU). SPRR1β and cathelicidin (CAMP) mRNA were quantitated using RT^2^ qPCR primer assays for SYBR® Green human SPRR1β and human CAMP from SABiosciences (Frederick, MD).

### Cytokine/Chemokine Assays

CXCL8 protein in medium from lower compartments of wells was measured using a sandwich ELISA [11]. The samples were assayed in duplicate and the results expressed in pg/mL, with an assay sensitivity of 3–6 pg/mL. CXCL10, CCL5, IL-29 and IL-6 were measured by multiplex ELISA (Milliplex® MAP Kit, Millipore Corporation, Billerica, MA), with samples assayed in duplicate. Assay sensitivity for CXCL10, CCL5, and IL-6 was 3 pg/mL, and 195 pg/mL for IL-29. The type III interferon, IL-29, was selected for measurement because it is the predominant interferon detected in this system. In preliminary experiments, IFN-β was present only at the threshold of detection, and in a minority of samples.

### Statistical Analysis

Thicknesses of cell layers were compared using paired t-test and Wilcoxon Signed Rank test using software (SigmaStat, Systat Inc.). SPRR1β and CAMP gene expression was analyzed by ΔΔCT method, with gene expression normalized to β-actin, and statistical analyses were done using paired t-test (SigmaStat). Treatment groups in RV16 replication experiments were compared via t-test or Mann-Whitney Rank Sum, also using software (SigmaStat). Effects of different levels of 1,25(OH)_2_D on cytokine/chemokine production in RV-infected cells were analyzed using three way ANOVA (Sigmastat).

## Results

### 1,25(OH)_2_D and RV Replication and RV-induced Cytokine Secretion

**Figure 1:**
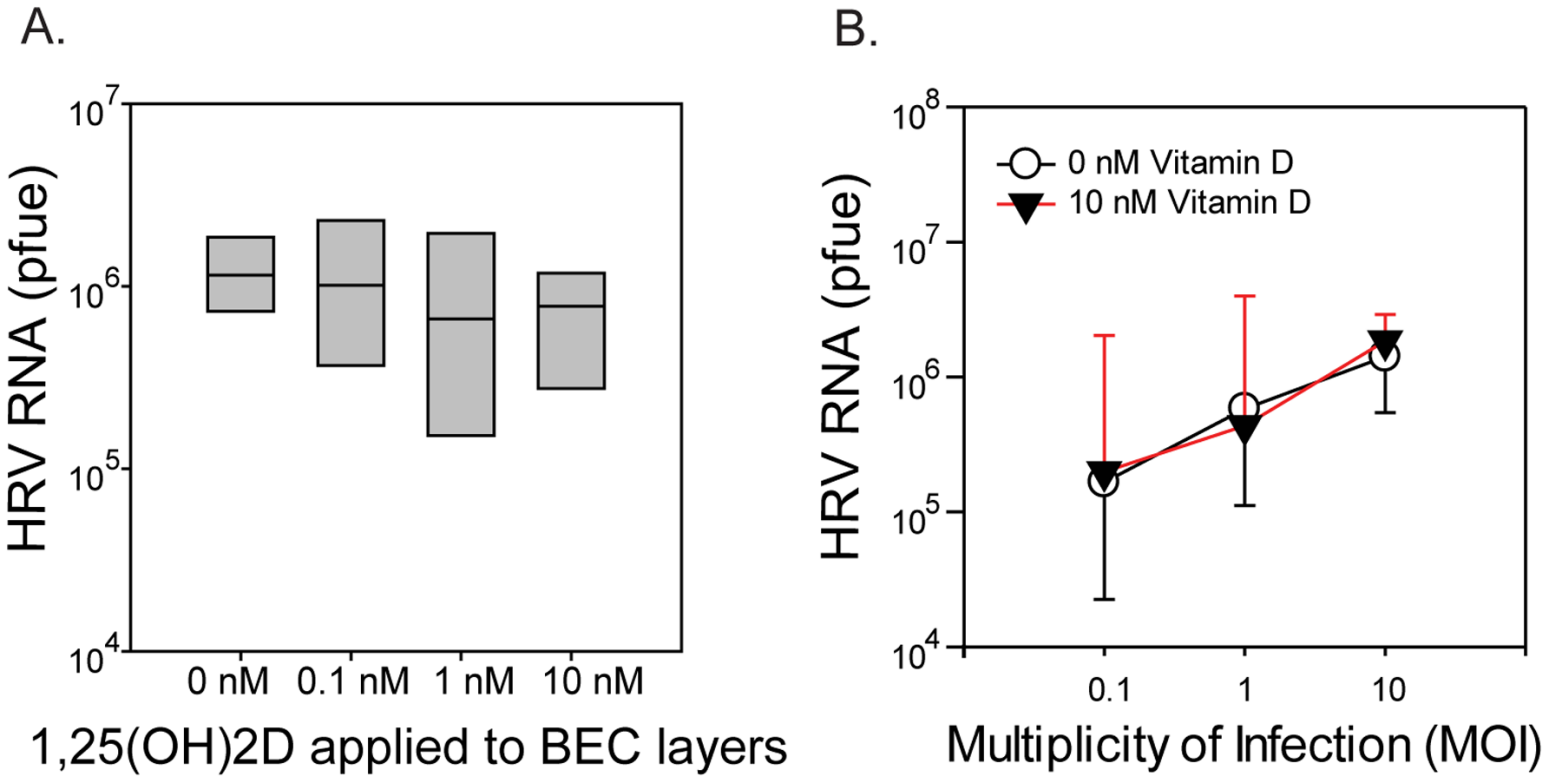
Effects of 1,25(OH)_2_D on RV replication. (A) Replication of RV16 at 24 hours in differentiated hBEC cultured at air-liquid interface for 24 days in the presence of various concentrations of 1,25(OH)_2_D (n = 7). (B) Replication of RV16 (MOI = 0.1, 1, or 10) at 24 hours in differentiated hBEC cultured at air-liquid interface for 27 days in the presence of 10 nM of 1,25(OH)_2_D (n = 3).

**Figure 2:**
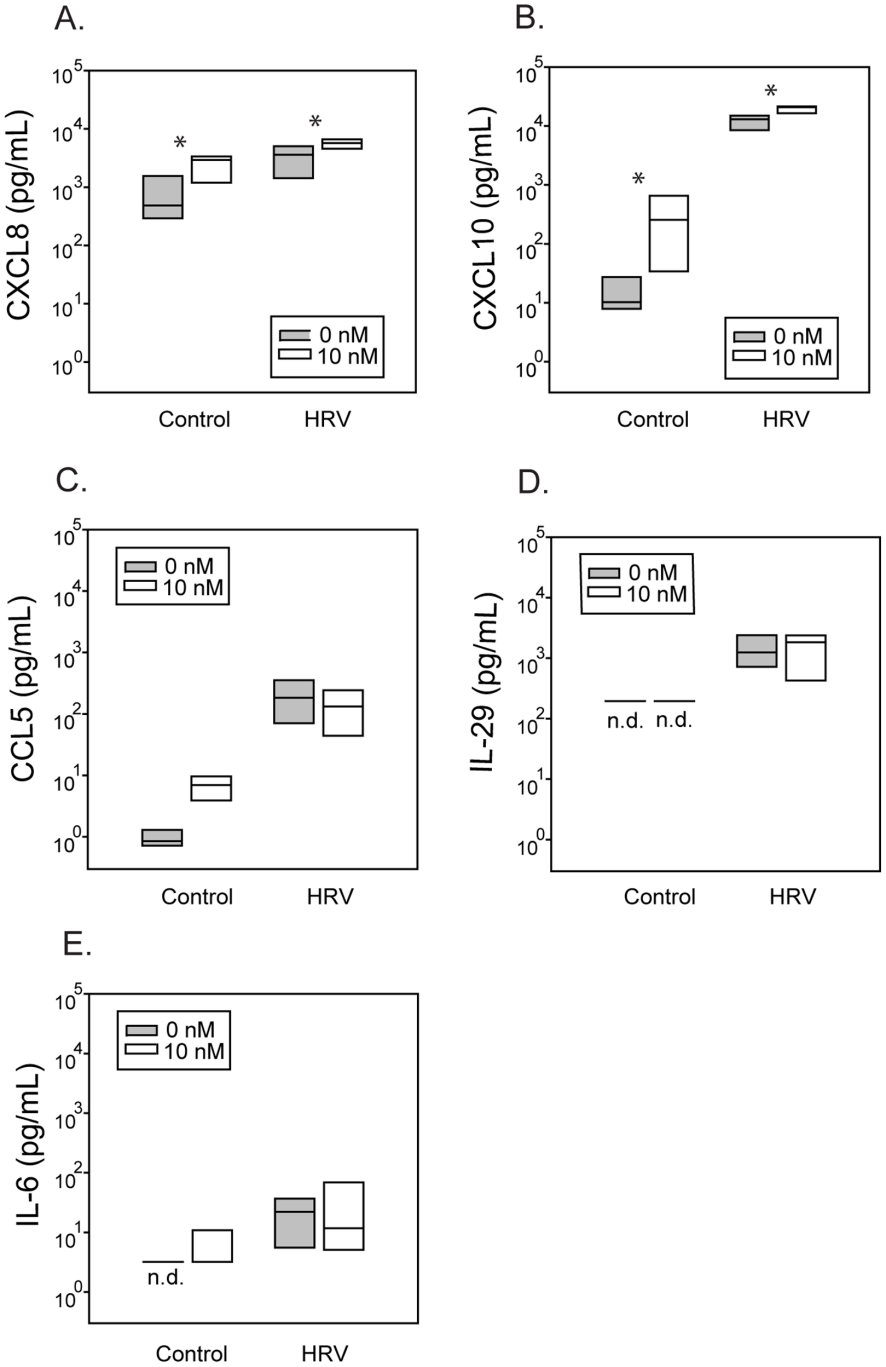
Effects of 1,25(OH)_2_D on cytokine secretion. (A,B) 1,25(OH)_2_D enhanced RV-induced CXCL8 (n = 6), and CXCL10 (n = 5) secretion. (C–E) Production of CCL5, IL-29, and IL-6 (n = 6) in these same cells, with “n.d.” indicating non-detectable levels. *p≤0.05 for cells treated with 1,25(OH)_2_D compared to untreated cells.

To determine whether vitamin D inhibits RV replication in epithelial cells, hBECs differentiated in the presence of 0, 0.1, 1, and 10 nM 1,25(OH)_2_D were infected with RV16 (MOI = 10) for 24 hours. Treatment of hBECs with 1,25(OH)_2_D had no significant effect on the replication of RV16 (Fig. 1A). This was true even at lower inoculating doses of virus (MOI of 0.1 and 1, Fig. 1B). RV16 induced CXCL8 (p<0.01) and CXCL10 (p<0.001). In addition, 1,25(OH)_2_D treatment enhanced secretion of CXCL8 (p<0.03) and CXCL10 (p<0.03) production, either in the presence or absence of infection (Fig. 2A, 2B). While RV infection stimulated increased production of CCL5, IL-6, and IL-29 by differentiated epithelial cells, treatment with 1,25(OH)_2_D did not alter this effect (Fig. 2C, 2D, 2E).

### Effects of Vitamin D on Cell Morphology and Transepithelial Resistance

**Figure 3:**
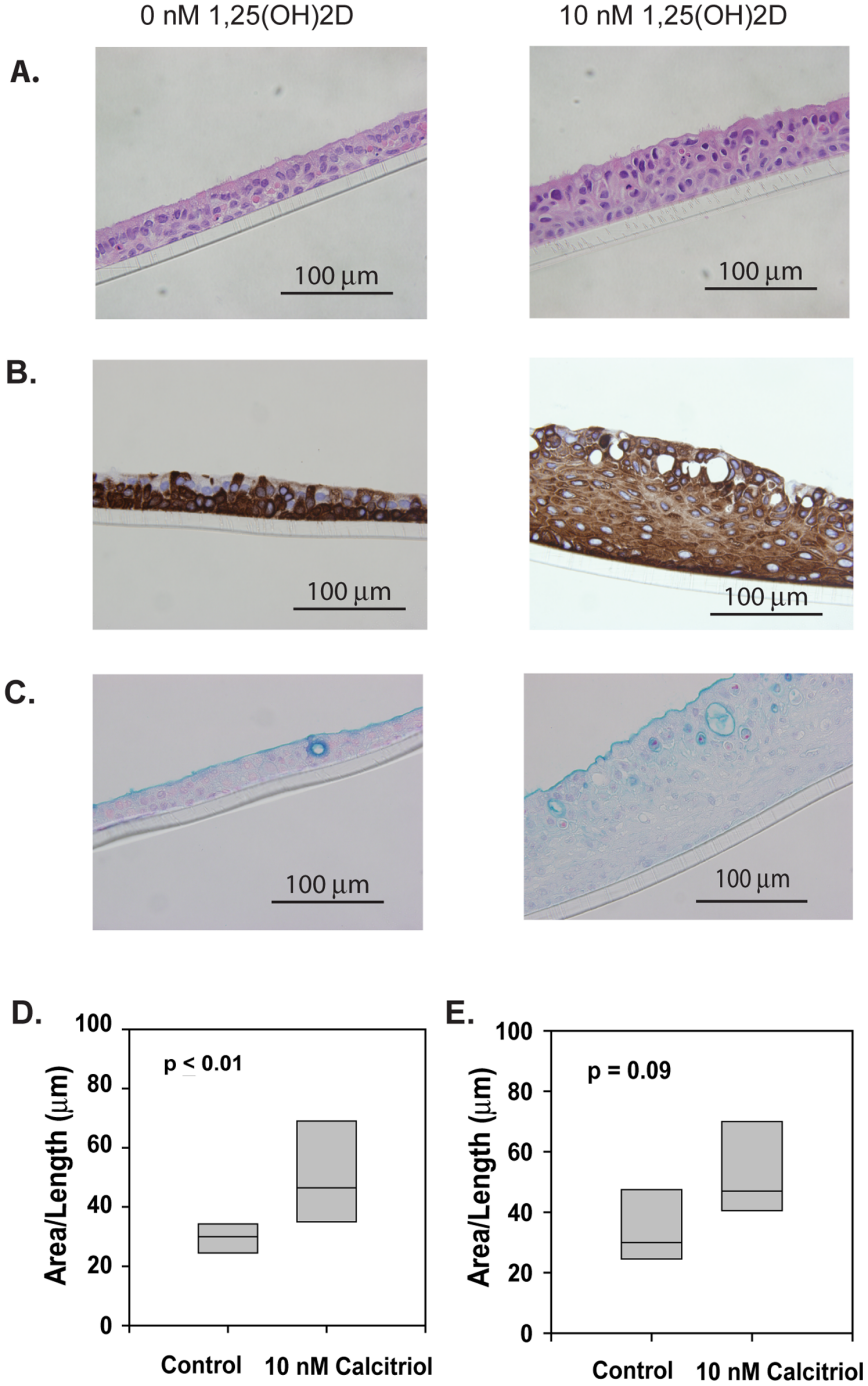
Effects of 1,25(OH)_2_D on cell morphology. (A) Hematoxylin and eosin stained paraffin cross-sections of primary hBEC layers cultured at an air-liquid interface for 24 days in the presence of 10 nM 1,25(OH)_2_D. (B) Staining of paraffin cross-sections from 1,25(OH)_2_D-treated primary hBEC cell layers with antibody against cytokeratin 5/6 (CK5/6). (C) Alcian blue stained paraffin cross-sections of primary hBEC layers cultured at an air-liquid interface for 24 days in the presence of 10 nM 1,25(OH)_2_D. (D) Average thicknesses of primary hBEC layers differentiated in the presence of 10 nM 1,25(OH)_2_D for 4 to 6 weeks (n = 8). (E) Average thicknesses of fully differentiated primary hBEC layers after treatment with 10 nM 1,25(OH)_2_D for 6 weeks (n = 5).

While preparing differentiated cells for the RV inoculation experiments, we noted that adding 1,25(OH)_2_D to the cultures led to thickened cell layers by day 25 (Fig. 3A). In addition, vitamin D also altered cell morphology, and some flattened cells at the base of the cell layer were observed. These sections stained negative for smooth muscle actin, vimentin and fibroblast specific protein, and apical cells stained positive for acetylated alpha tubulin; a robust ciliated cell marker (data not shown). Enhanced expression of cytokeratin 5/6, a basal cell marker, was seen in the subapical cells (Fig. 3B). Alcian blue staining revealed the presence of increasing numbers of mucin-filled cysts with higher levels of 1,25(OH)_2_D (Fig. 3C). These changes became so exaggerated in cell layers exposed to the upper limit of the concentration range (100 nM) as to create a dysmorphic appearance. Treatment with 10 nM 1,25(OH)_2_D caused significant thickening whether it was added during differentiation (Fig. 3D) or after differentiation (Fig. 3E).

**Figure 4:**
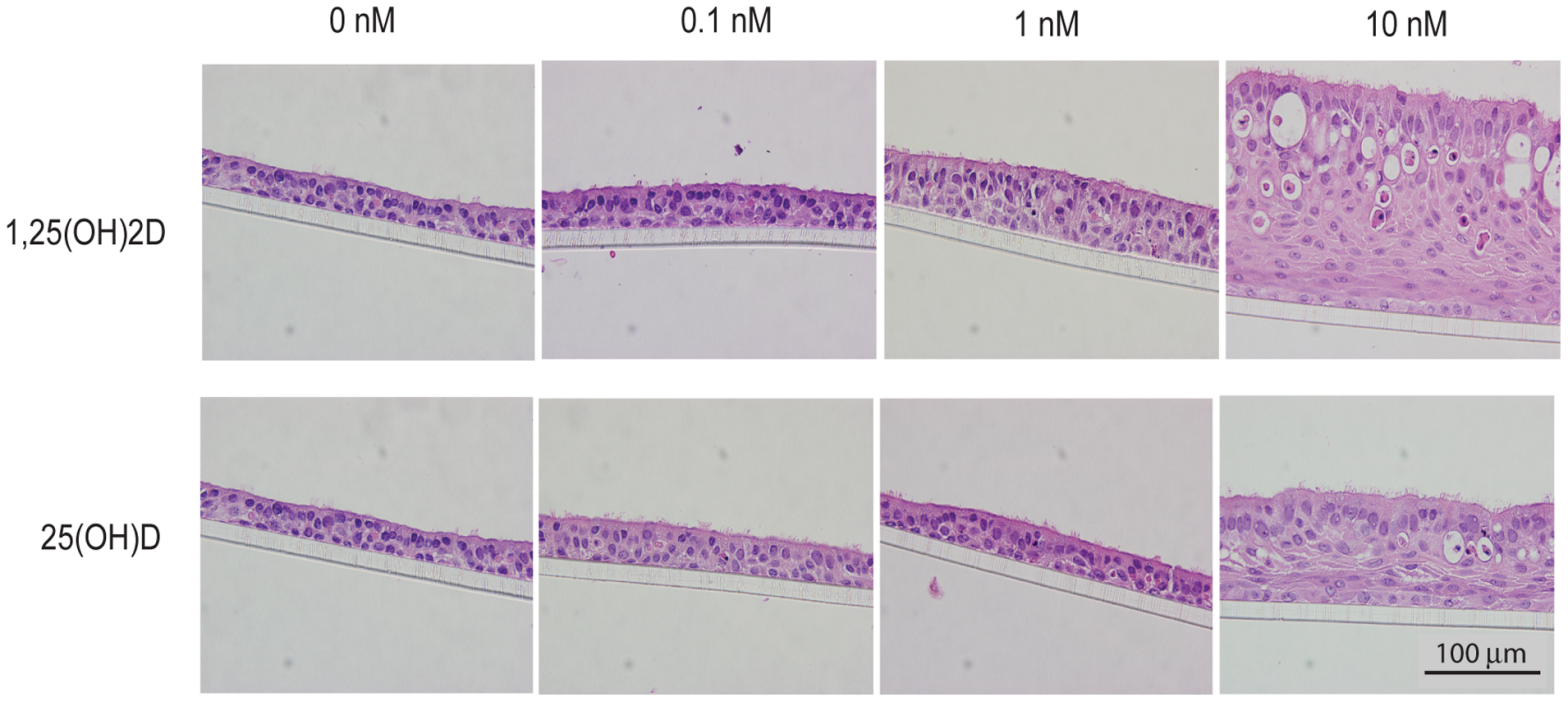
1,25(OH)_2_D and 25(OH)D have similar effects on cell growth and differentiation. Hematoxylin and eosin stained paraffin cross-sections of primary hBEC layers cultured at an air-liquid interface for 28 days in the presence of various concentrations of 1,25(OH)_2_D or 25(OH)D.

Respiratory epithelial cells can express 1α-hydroxylase [1], suggesting that the vitamin D precursor 25(OH)D and the active hormone should have similar effects on cell morphology. To test this theory, we treated differentiating hBEC cultures with 0, 0.1, 1, and 10 nM of either 1,25(OH)_2_D or 25(OH)D over the course of 21 days. Both forms of vitamin D caused similar thickening of the hBEC layers (Fig. 4).

Notably, culturing hBEC in the presence of 0, 0.1, 1 or 10 nM 1,25(OH)_2_D had no effect on transepithelial resistance (data not shown), an indicator of tight junction formation. Transepithelial resistance was somewhat lower for cell layers treated with 100 nM of 1,25(OH)_2_D, likely a function of the dysmorphic changes noted previously. Time until cilia development also was not affected by 1,25(OH)_2_D treatment, with cilia detected between day 17 and 21 as is typical in this culture system (data not shown). Finally, vitamin D did not affect healing of differentiated cells after mechanical wounding (data not shown).

### Expression of SPRR1β and Cathelicidin

To further characterize the nature of the cells in the thickened cell layers of calcitriol-treated cultures, expression of small proline-rich protein 1β (SPRR1β; also “cornifin”), a marker of squamous metaplasia [12]–[14], was examined via quantitative PCR. Treatment of fully differentiated cultures with 10 nM 1,25(OH)_2_D for 6 weeks caused a 14-fold upregulation of SPRR1β expression (p<0.05), suggesting that the cells making up the bulk of the thickened layers were squamous in nature. As a positive control, we also measured expression of cathelicidin (CAMP/hCAP18/LL-37), a vitamin D-induced antimicrobial peptide [15]. Treatment of epithelial cells with 10 nM 1,25(OH)2D either during or after differentiation significantly induced SPRR1β (Table I), and similar trends were noted for cathelicidin.

### Vitamin D vs. Vitamin A Effects on Differentiation of hBECs

**Figure 5:**
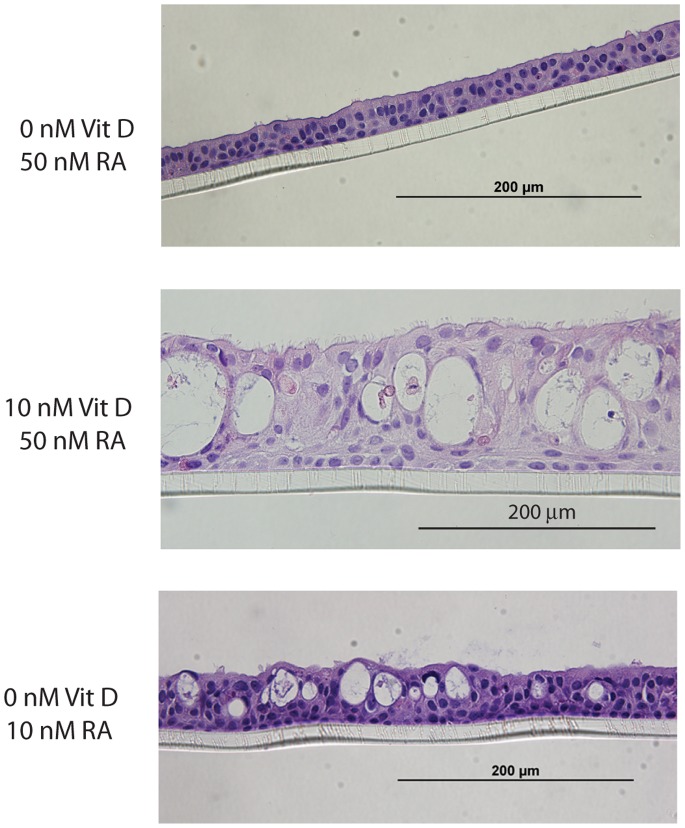
Similar effects of supplemental vitamin D vs. retinoic acid deficiency on cell morphology. Hematoxylin and eosin-stained paraffin cross-sections of primary hBEC layers cultured at an air-liquid interface for 28 days in the presence of 10 nM (deficient) or 50 nM (normal) all-*trans* retinoic acid, with 0 nM or 10 nM 1,25(OH)_2_D.

The vitamin D receptor (VDR) shares a common subunit (retinoid X receptor, “RXR”) with the vitamin A receptor (RAR), suggesting that vitamin D could interfere with vitamin A-induced cell differentiation [16], [17]. To examine possible interaction between the two compounds on epithelial cell differentiation, hBECs were differentiated in the presence of increasing levels of 1,25(OH)_2_D and all-*trans* retinoic acid (vitamin A). Retinoic acid is required for epithelial cell differentiation [18], and the layers failed to successfully differentiate with 0 nM and 1 nM retinoic acid, regardless of the level of 1,25(OH)_2_D that was present. Interestingly, cells that were differentiated in the presence of a suboptimal level (10 nM) of retinoic acid (and without vitamin D) exhibited some of the same morphological features (thickened layers, mucous cysts) as those differentiated in the presence of increased levels of 1,25(OH)_2_D (Fig. 5). For example, treatment with 10 nM vs. 50 nM retinoic acid caused increased epithelial thickness (median of 35.5 µm [26.7–40.7] vs. 27 µm [24.5–31.7], p<0.05). In addition, RA-deficiency induced SPRR1β mRNA (7-fold increase, p<0.01) and a there was a nonsignificant trend for increased cathelicidin (Table 1).

**Table 1:** Effects of 10(fold upregulation).

*Gene*	10 nM CalcitriolDuring Differentiation	10 nM CalcitriolPost-differentiation	Low RA
	(n = 6)	(n = 5)	(n = 5)
***SPRR1β***	6.3 (1.7–12.4)**	14.2 (1.9–36.7)*	7.3 (3.3–13.1)**
***CAMP***	5 (0.7–10.2)	9.9 (2.7–21.1)**	3 (0.6–6.3)

* p<0.05.

** p≤0.01.

## Discussion

Vitamin D status has been inversely associated with the prevalence of common colds [6], [7], suggesting the possibility that it inhibits viral replication or the induction of pro-inflammatory cytokine response. Here, we have demonstrated that treatment of differentiating primary hBECs with vitamin D in the form of either 25(OH)D (pre-hormone) or 1,25(OH)_2_D (active hormone) had no direct effects on RV replication, even after low-dose inoculation. Vitamin D had small but significant effects on epithelial cell chemokine production, but did not affect RV-induced secretion of the type III interferon IL-29. These findings suggest that associations between vitamin D levels and the frequency or severity of cold infections are not due to a direct antiviral effect related to RVs.

It is of note that Hansdottir et al conducted experiments in airway epithelial cell monolayers to determine whether vitamin D inhibited replication of respiratory syncytial virus (RSV), an enveloped RNA virus [19]. Similar to our results, vitamin D did not affect RSV replication. In contrast to our results, the authors found that vitamin D inhibited induction of certain pro-inflammatory cytokines and chemokines, including IL-29. The discrepant results could be due to the use of different viral pathogens, or use of a monolayer cell culture system as opposed to a multi-layered, differentiated system.

We noted that vitamin D had obvious effects on epithelial cell growth and differentiation. Vitamin D produced marked changes in cellular morphology and increased expression of markers of basal cells (cytokeratin 5) and squamous metaplasia (SPRR1β). Notably, effects on growth and differentiation were similar when the cells were treated with 25(OH)D or 1,25(OH)_2_D. This finding is consistent with recent reports that respiratory epithelial cells convert inactive vitamin D to its active form [1]. This finding has important implications since circulating levels of 25(OH)D are approximately 100-fold higher than the active form of the hormone. We used 0.1–100 nM vitamin D in our experiments, which is in the same range as optimal circulating 25(OH)D serum levels (30–80 ng/mL; 75–200 nM). Levels of vitamin D in airway fluids are unknown. In healthy airways, vitamin D levels may be considerably lower than in the bloodstream. Airway inflammation is associated with an influx of serum proteins [20], and under these conditions vitamin D levels could be considerably higher. This prompted us to evaluate potential effects of vitamin D on wound repair, however, the findings showed no significant effects on this process.

While the vitamin D-treated airway epithelial cell layers in our system became noticeably thicker, data from previous studies demonstrated that vitamin D can inhibit smooth muscle cell proliferation [21]. Incidentally, we found in preliminary experiments that both 25(OH)D and 1,25(OH)_2_D had a mild inhibitory effect on growth of undifferentiated airway epithelial cell monolayers (data not shown). Collectively, these findings suggest that effects of vitamin D on cell growth may depend on the type of cell and differentiation state.

A recent study found that vitamin D deficiency in mice inhibited lung growth and caused deficits in lung function [22]. It joins a prior body of work that indicates vitamin D deficiency can affect lung development via multiple mechanisms: structural, functional, and immunological, both *in utero* and postnatally [23]. When considered together with these findings, our results support the concept that vitamin D could affect epithelial growth and differentiation during periods of human lung growth and development.

Vitamin D is part of a complex system of interacting elements, and other agents, such as vitamin A, can modulate its effects [17]. These two vitamins, when bound to their respective receptors, form heterodimers with a shared partner protein (retinoic X receptor), and our experiments suggest that vitamins A and D may have opposing effects on epithelial cell differentiation. Similarly, vitamins A and D can have opposing effects on the skeletal system, with high levels of retinoic acid interfering with the vitamin D-regulated maintenance of normal serum calcium levels [16]. As a result, high vitamin A intake is associated with an increased incidence of osteoporosis and hip fractures [24], [25]. Our findings suggest that excessive amounts of vitamin D in the airway could have detrimental effects on epithelial cell differentiation, especially if vitamin A status is marginal. In this situation, cell layers exhibit dysmorphic changes, including reduced transepithelial resistance and squamous metaplasia, a mechanism that has been associated with airway pathology in smoking [26].

Vitamin D levels have been inversely related to the frequency and severity of respiratory illnesses including influenza and the common cold [6], [7], [27], [28]. Protective effects of vitamin D against bacteria and certain enveloped viruses such as influenza virus are presumed to occur through increased expression of antimicrobial peptides such as cathelicidin, which is secreted by epithelial cells into the airways fluid [29], [30]. This protein, upon binding to microbial membranes, changes membrane permeability through a pore-forming mechanism, thus disrupting metabolism and destroying the microbe [31], [32]. In contrast to enveloped viruses and bacteria, RVs have a protein capsid, and we found no evidence that vitamin D treatment inhibited RV replication. Our findings suggest that the inverse association between vitamin D status and cold frequency could be due to indirect mechanisms [8], [33]. For example, vitamin D could reduce colonization with bacterial pathogens (e.g. *S. pneumoniae*) that might synergize with viruses to produce more severe respiratory illnesses [34].

Strengths of this study include the use of primary cultures of airway epithelial cells, with consideration of vitamin D effects on cells grown at air-liquid interface. Effects on growth and morphology were reproducible using the cells from multiple primary cell donors. It is also important to consider that our model lacks immunologically important accessory cells, such as neutrophils, eosinophils, and macrophages, all of which could contribute to antiviral defenses in vivo. Thus, while vitamin D had no direct antiviral effect on epithelial cells, there could be indirect antiviral effects mediated by other cells. Since epithelial cells can convert 25(OH)D into 1,25(OH)_2_D, they could serve as a source of the active hormone for other cells in the airway microenvironment.

Vitamin D has been linked to reduced respiratory illnesses in several studies, but the mechanisms for these effects are unclear. Our findings demonstrate that vitamin D did not have direct anti-RV effects in epithelial cells, but could affect the quality of the antiviral immune response by inducing CXCL8 and CXCL10. Incidentally, we found that vitamin D also affects epithelial cell growth and differentiation, especially if vitamin A status is marginal.
